# Supplementary material for: Spatial kinetics and immune control of murine cytomegalovirus infection in the salivary glands
Source: PLoS Comput Biol. 2024 Aug 16;20(8):e1011940. doi: 10.1371/journal.pcbi.1011940 (PMC11357091; doi:10.1371/journal.pcbi.1011940)
Supplement: S1 Text — (PDF) [file pcbi.1011940.s001.pdf]

Supporting Information for: Spatial kinetics and immune control of murine cytomegalovirus infection in the salivary glands

Catherine M Byrne[1], Ana Citlali Márquez[2], Bing Cai[3], Daniel Coombs[4], Soren Gantt[5*]

[1] Vaccine and Infectious Disease Division, Fred Hutchinson Cancer Center, Seattle, Washington, United States of America
[2] British Columbia Centre for Disease Control, Vancouver, British Columbia, Canada
[3] British Columbia Children's Hospital Research Institute, Vancouver, British Columbia, Canada
[4] Department of Mathematics, The University of British Columbia, Vancouver, British Columbia, Canada
[5] Département de Microbiologie, Infectiologie et Immunologie, Université de Montréal, Montréal, Québec, Canada.

*Corresponding author
Email: soren.gantt@umontreal.ca

Supplementary Figures

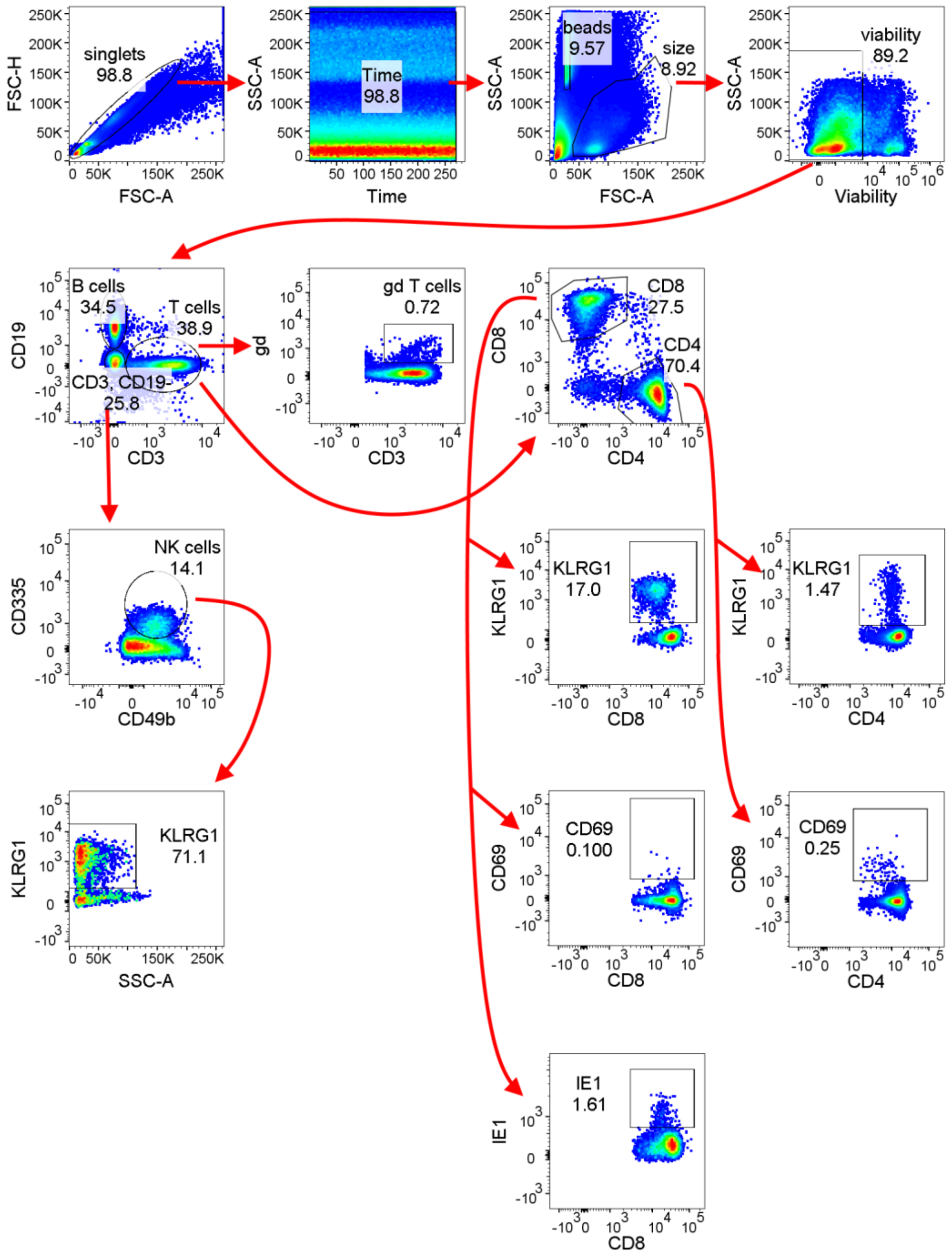

Fig A: Gating strategy used to identify immune cell populations of interest. Cells were first gated against FSC-H and FSC-A to remove doublets, then against time and SSC-A to ensure no acquisition issues. We further gated against FSC-A and SSC-A to identify cells of the appropriate size, and against SSC-A and the viability dye used to identify live cells. Live cells were then gated using remaining markers to identify the cell populations of interest.

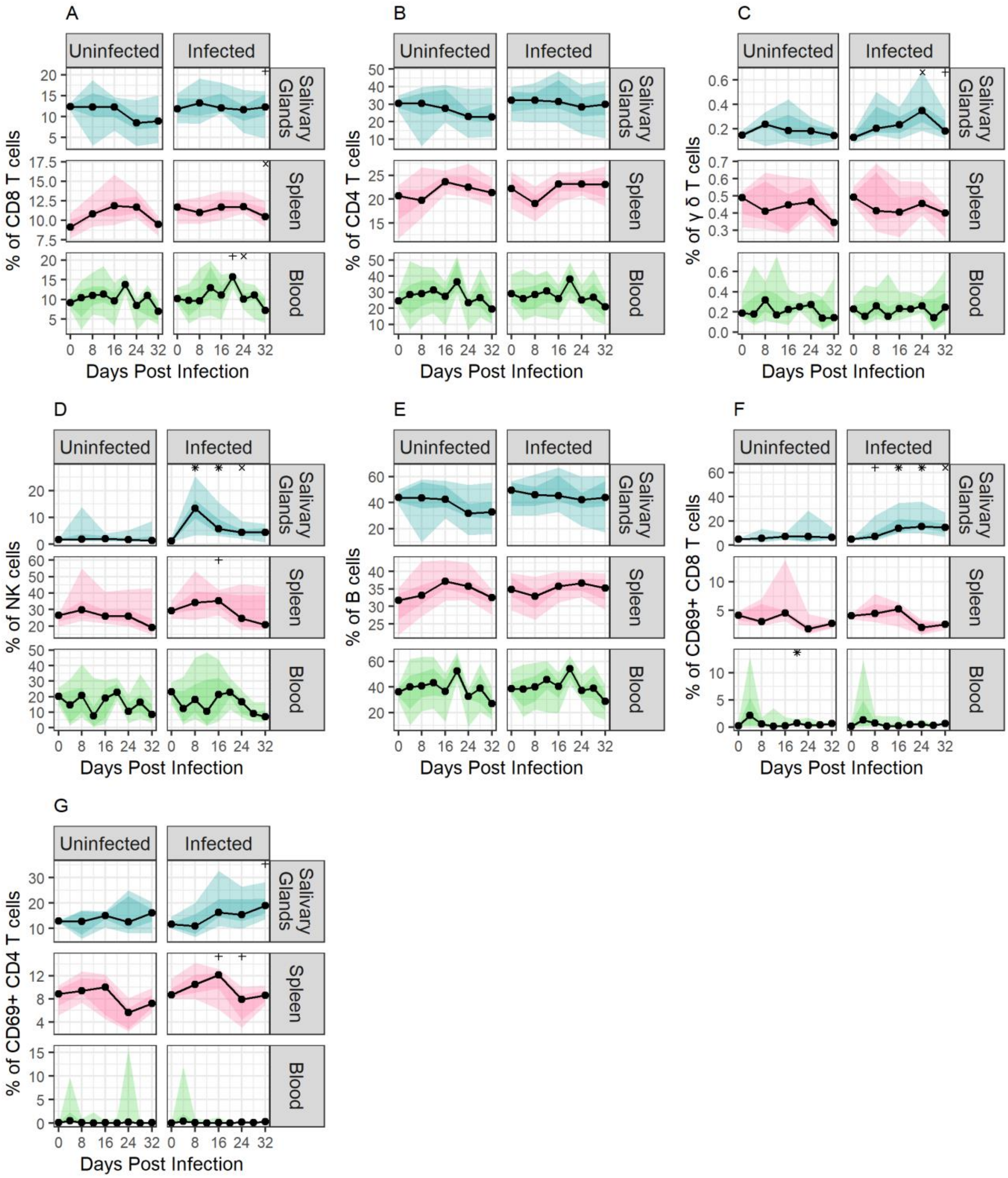

Fig B: Immune cell populations of secondary interest and their change over the course of observation in uninfected and MCMV-infected mice. Symbols +, ×, and * above data indicate days where an immune cell proportion was significantly different between uninfected and infected mice. Symbol "+" represents where the p-value was less than 0.05, symbol "×" represents where the p-value was less than 0.005, and symbol "*" represents where the p-value was less than 0.0005. The symbol position is always above the group that had a higher median value than its comparator. Plots A-C are reported as the percentage of viable cells while D-G are reported as percentage of parent population.

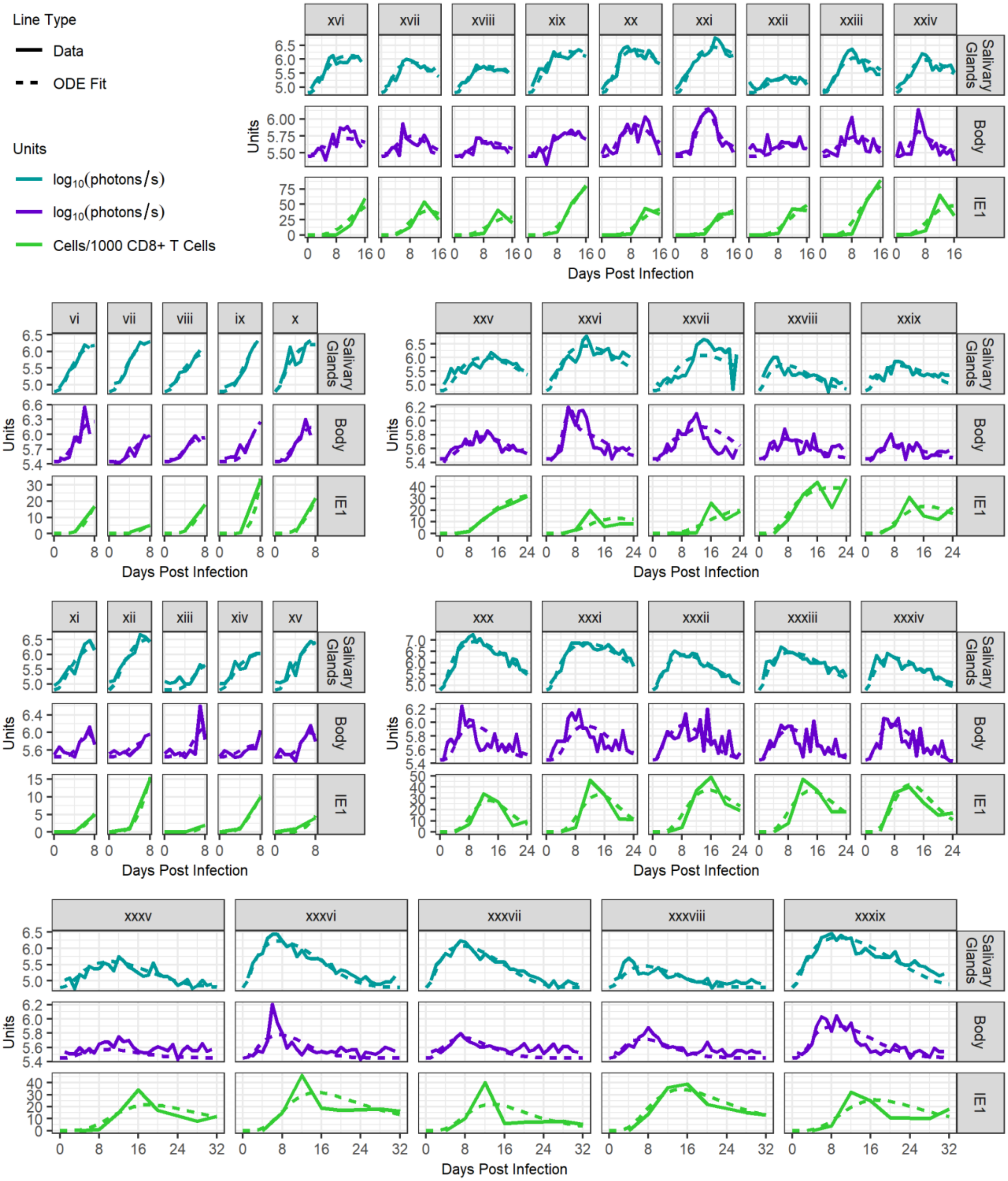

Fig C: Additional fits to mice infected ISG with 1000 PFU K181-luc.

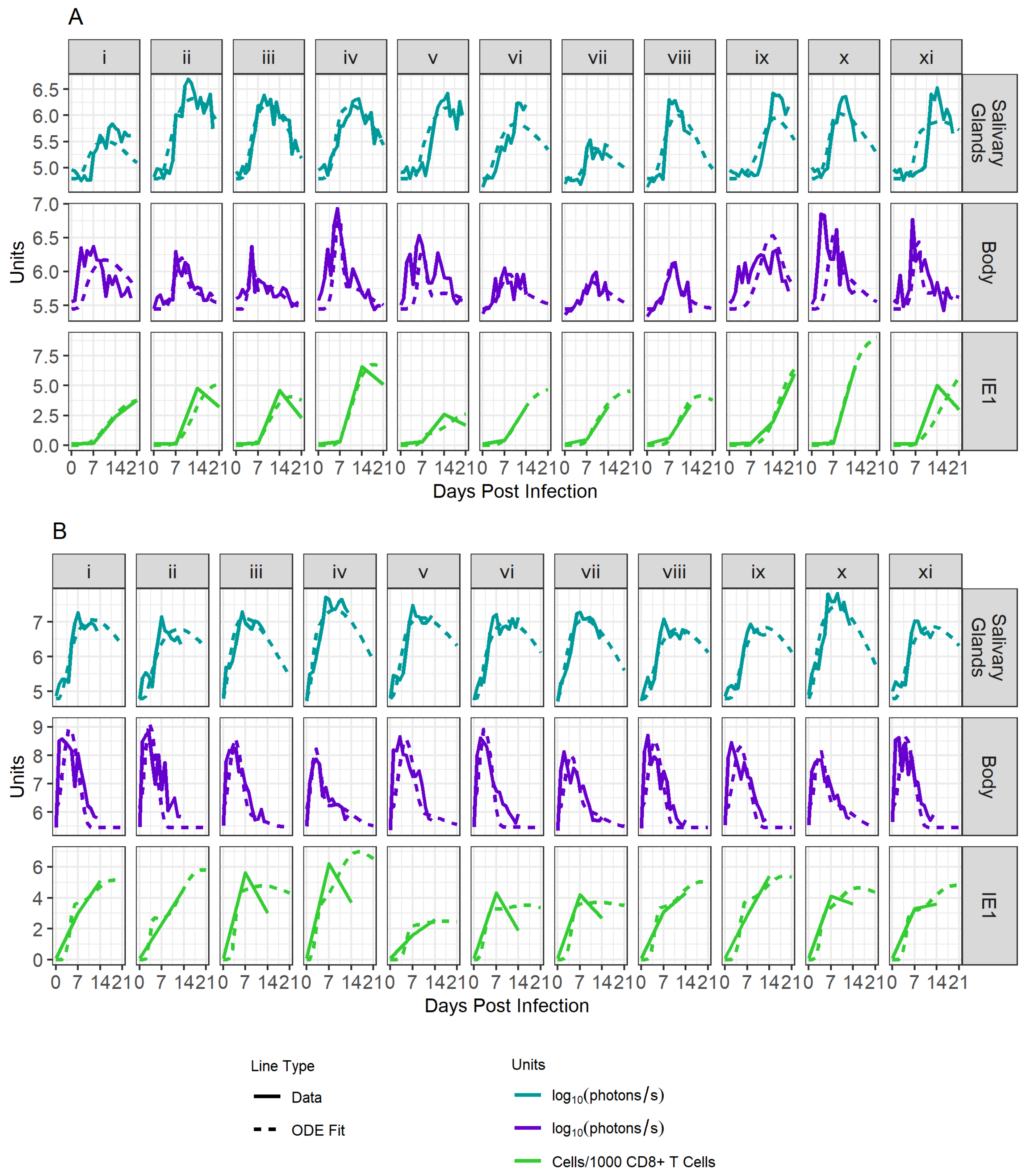

Fig D: Fits to mice infected IP with K181-luc. Panel A shows model fits for data from mice infected with 100 PFU while panel B shows model fits for data from mice infected with 1,000,000 PFU.
